# Characterization of intraocular immunopathology following intracameral inoculation with alloantigen

**Published:** 2008-03-26

**Authors:** Daniel R. Saban, Ian A. Elder, Cuong Q. Nguyen, W. Clay Smith, Adrian M. Timmers, Maria B. Grant, Ammon B. Peck

**Affiliations:** 1 Department of Pathology, Immunology and Laboratory Medicine; 2 Department of Ophthalmology; 3 Department of Pharmacology and Therapeutics; University of Florida, Gainesville, FL

## Abstract

Purpose: Anterior chamber-associated immune deviation (ACAID) is a form of peripheral tolerance achieved via intracameral antigen inoculation. It is well known that ACAID effectively down-regulates potentially destructive immunities such as delayed-type hypersensitivity (DTH) at extraorbital sites. However, what has not been specifically addressed is whether local intraocular tissues are negatively affected from intracamerally placed antigen. Thus, the current study was undertaken to detect and characterize potential pathological effects on intraocular tissues following intracameral inoculation with alloantigen.

Methods: ACAID induced in C57BL/6 hosts via intracameral inoculation with allogeneic (BALB/c) splenocytes was confirmed by the absence of DTH reactivity in the periphery. Injuries to the anterior segment and neuroretina following intracameral inoculation were evaluated pathologically via histological evaluation, molecularly via upregulation of glial fibrillary acidic protein (GFAP), and functionally via assessment of photoreceptor degeneration and electroretinogram (ERG) out to 24 days. In all experiments, intracamerally inoculated mice were compared to sham-operated, and controlled lens-punctured mice—a procedure that elicits intracameral inflammation for positive identification of immunopathological changes.

Results: Inflammation of anterior segment tissues persisted out to eight days, despite evidence that significant clearance of allogeneic cells took place within 6 h. In the neuroretina, a transient loss in ERG B-wave amplitudes was detected, but photoreceptor degeneration and GFAP upregulation were not.

Conclusions: Intracameral inoculation with alloantigen leads to anterior segment inflammation and ERG dysfunction; however, this was markedly reduced and transient when compared to strong anterior segment inflammation induced by a more serious lens-puncture wound.

## Introduction

For more than a century, scientists have been fascinated by the immune privileged status of the eye, particularly the prolonged survival enjoyed by foreign tissue grafts placed intracamerally, which would otherwise be rejected at extraocular sites [1-3]. It is currently understood that ocular immune privilege is a means to protect visual acuity from deleterious immune responses and involves numerous distinct mechanisms. Examples of these mechanisms include a restrictive blood-ocular barrier, lack of lymphatic drainage networks, absence of molecules involved in antigen presentation, such as major histocompatibility complex (MHC) class II, expression of molecules involved in immunomodulation, such as Fas-FasL, tumor necrosis factor–related apoptosis-inducing ligand (TRAIL), and B7.2, and the presence or secretion of immunoregulatory factors, such as α-melanocyte stimulating hormone, thrombospondin, and transforming growth factor-β (TGF-β) [1,4-12].

Kaplan and Streilein were the first to describe anterior chamber-associated immune deviation (ACAID), a peripheral form of immunological tolerance induced by experimental injection of antigen delivered intracamerally [2,3]. This form of tolerance is mediated by a heterogeneous population of regulatory T cells in the spleen which selectively inhibit the activation/expansion of (1) pathogenic effector T cell populations involved in delayed-type hypersensitivity (DTH) responses; and (2) B cell populations involved in production of complement-fixing antibodies [13-20]. ACAID has therefore continued to receive considerable attention in several areas of research. One potentially important area involves the ability of ACAID to effectively suppress an array of immunopathological conditions in rodent models such as skin and corneal allograft rejection, autoimmune encephalomyelitis and uveoretinitis, and autoimmune airway hyperreactivity [2,3,21-23]. Alternatively, ACAID induction is also used as an experimental readout to assess ocular immune privilege and integrity in models of corneal transplantation and neovascularization, uveitis, and glaucoma [24-26].

To date, only few antigens (e.g, *L. monocytogenes*, and certain tumor cell lines) have been reported that circumvent ACAID induction and elicit deleterious inflammation following intracameral inoculation [27-29]. Indeed most antigens tested (e.g., ovalbumin (OVA), bovine serum albumin (BSA), or alloantigen) result in the induction of ACAID following intracameral inoculation. However, whether such antigens can be pathogenic to intraocular tissues during this process has not been adequately addressed. In the current study, we determined whether inoculation with alloantigen, which is known to induce ACAID, causes pathological injury of intraocular tissues in the process. To this end, we comprehensively evaluated the integrity of intraocular tissues following intracameral inoculation via histological, molecular, and functional examination. We used a novel approach which directly compared intracamerally inoculated mice to controlled lens-punctured mice. The latter is an experimental procedure known to elicit robust intracameral inflammation and thereby incorporated here as a means to positively identify immunopathological changes within the eye [30-33]. Sham-operated mice were also included. Based on this analysis, we found that intracameral inoculation with alloantigen leads to anterior segment inflammation and (electroretinogram (ERG) dysfunction; however, this was markedly reduced and transient when compared to strong anterior segment inflammation induced by a more serious lens-puncture wound.

## Methods

### Animals and anesthesia

All animals were treated according to guidelines established by the ARVO Statement for the Use of Animals in Ophthalmic and Vision Research and the Public Health Policy on Humane Care and Use of Laboratory Animals (U.S. Public Health Review). Female C57BL/6, C57BL/6.eGFP, and BALB/c mice six to eight weeks of age (Jackson Laboratories, Bar Harbor, Maine) used for these experiments were maintained under 12h:12h light-dark cycles. Anesthesia was performed on mice with an intraperitoneal (IP) injection of 100 μl PBS containing 3 mg ketamine and 0.0075 mg xylazine.

### Intracameral inoculation and lens puncture

Cell suspensions used for intracameral inoculations in C57BL/6 hosts were prepared by pressing harvested BALB/c spleens through a sterile meshing. Erythrocytes were lysed by incubation for 7 min in a 0.15 M solution of NH_4_Cl at room temperature, and thoroughly washed. Remaining splenocytes were counted using a hemacytometer and diluted to appropriate concentrations for injections, which was 1x10^6^ BALB/c splenocytes in 2 ul sterile Hank’s balanced salt solution (HBSS).

**Figure 1:**
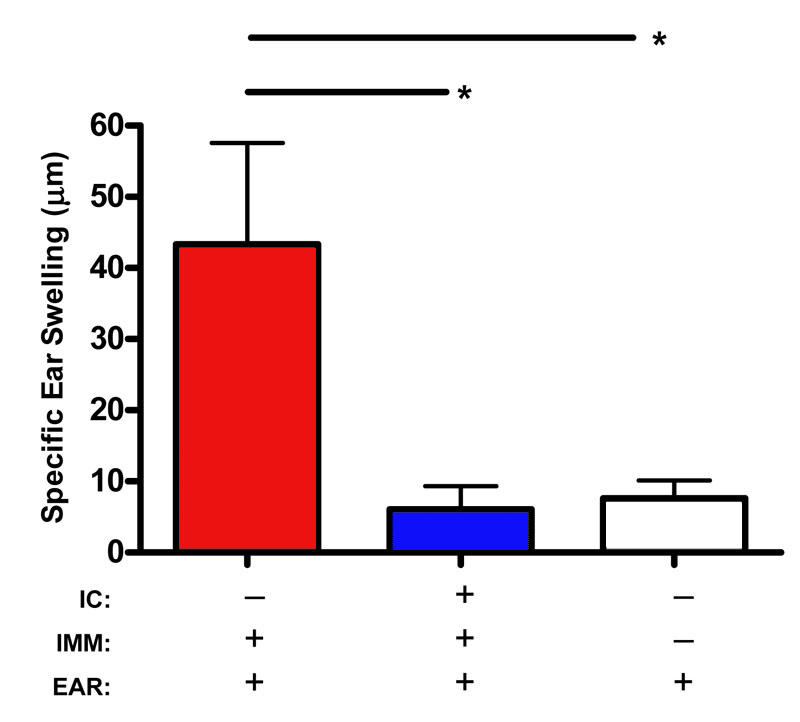
Verification of anterior chamber-associated immune induction in C57BL/6 hosts to BALB/c alloantigen. C57BL/6 hosts were intracamerally (IC) inoculated on day 0 with BALB/c allosplenocytes. This was followed by an allomatched immunization (IMM) on day 7, and an allomatched intrapinnal ear (EAR) challenge on day 14 (n=7). In addition, to identify positive delayed type hypersensitivity (DTH) following ear challenge a control group was immunized without prior IC inoculation (n=7); and a second control group (n=6) to identify the absence of DTH did not receive either IC or IMM. ANOVA was used to calculate the statistical significance. Asterisk (*) indicates p≤0.05.

Intracameral inoculation has been described previously [2,3]. Briefly, the corneal apex of deeply anesthetized mice was punctured parallel to the iris with a 30-gauge insulin syringe (Becton Dickinson and Co., Franklin Lakes, NJ), and the aqueous humor (AqH) tapped by gentle compression. Approximately 2 μl air was dispensed into the anterior chamber via puncture, followed by a 2 μl splenocyte suspension, or HBSS only as a sham to control for surgical trauma. In the experiment which measured clearance of alloantigen from the anterior chamber, BALB/c mice were used as hosts and allosplenocytes were instead harvested from C57BL/6.eGFP+ mice.

Anterior lens puncture was also included because this experimental procedure is a well documented method to elicit intracameral inflammation [30-33]. This was accomplished by transcorneal puncture at the corneal apex with a sterile 30-gauge needle, and the needle was then advanced into the lens to a depth of approximately 0.5 mm. Effective punctures were confirmed by slit-lamp biomicroscopy, and identified by cataract formation within three days.

### Delayed-type hypersensitivity assay

Splenocyte suspensions were irradiated (3000R) and set at a dose of 1x10^6^ cells/10 μl. Each dose was administered intrapinnally using a 1 ml insulin syringe (30 gauge), which was advanced toward the superior edge and dispensed accordingly. Ear thickness was measured using an engineer’s micrometer (Mitutoyo, Aurora, IL) at 3 time-points, which include: 1) prior to injection as a baseline; 2) 24 h post injection; and 3) 48 h post injection. At each of these time points, the mean of 3 independent measurements per ear was calculated. Specific ear swelling was then calculated by subtracting the mean baseline from the mean peak measurement (at 24 or 48 h) per ear.

**Figure 2:**
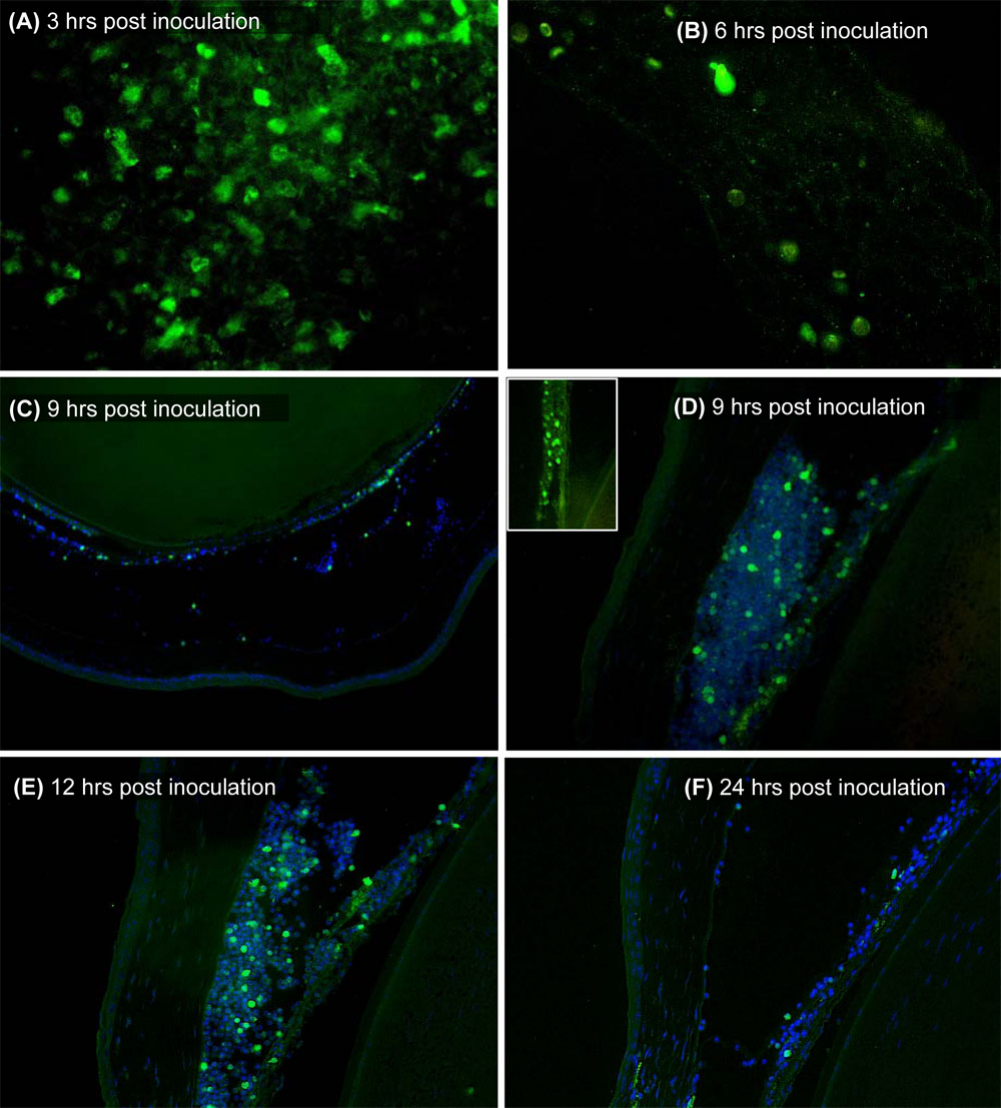
Allosplenocytes are rapidly cleared from host anterior chamber following intracameral inoculation. BALB/c eyes were enucleated at 3 to 24 h post intracameral inoculation (at least n=2 per micrograph) with enhanced green fluorescent protein (eGFP)+ allosplenocytes (green). Some sections were counterstained with DAPI (blue) for epifluorescence microscopy. Representative high-magnification images demonstrate a reduced presence of eGFP(+) observed in the central anterior chamber (AC) from 3 h **(A)** to 6 h **(B)** post inoculation. Representative low-magnification images demonstrate the few eGFP(+) cells observed in the central AC at 9 h **(C)**, while eGFP(+) cells were abundant in the iridocorneal angles **(D),** and similarly observed at 12 h **(E)**. Few eGFP(+) cells were also observed trafficking through the iris/ciliary body **(D, inset)**. Host AC were absent of eGFP(+) cells by 24 h, with the exception of few cells and cell fragments remaining in some of the inoculated mice **(F)**.

### Electroretinogram analyses

All mice, dark-adapted overnight, were anesthetized, and their eyes were treated with 0.5% proparacaine hydrochloride (Akorn, Inc., Lincolnshire, IL) for topical anesthesia and 2.5% phenylephrine hydrochloride (Bausch & Lomb, Rochester, NY) to induce mydriasis. Gold contact electrodes were placed on the corneas with 2.5% ophthalmic methyl cellulose (Akorn, Inc.). Reference and ground electrodes were placed subcutaneous on the head and hind leg, respectively. Full-field scotopic ERG analysis was acquired using a Toennies Multiliner Vision electrodiagnostic system with Ganzfeld stimulator (LKC Technologies, Gaithersburg, MA). Measurements were made on both the injected eye and the untreated contralateral control eye simultaneously at seven flash intensities ranging from 0.01 mcd⋅s/m^2^ to 5 cd⋅s/m^2^. The retinal responses at each of the flash intensities were measured 10 times and averaged. Following ERG analysis, eyes were coated with bacitracin-neomycin-polymyxin antibacterial ophthalmic ointment (Pharmaderm, Melville, NY). B-max values were obtained by fittings of the nonlinear saturating Naka-Rushton equation to the B-wave amplitudes [34].

### Identification of photoreceptor degeneration

Histological cross-sections containing the optic nerve were consistently used for counting the number of photoreceptor nuclei. Along the outer nuclear layer (ONL) within each section, nine equidistant reference points were identified so that the location of the first reference point was adjacent to the limbus, the central reference point overlapped the optic nerve, and the last point was adjacent to the opposing limbus. At each reference point, three random locations within the ONL were selected. For each random location, adjacent nuclei were counted within the ONL from the outer limiting membrane to the outer plexiform layer [35]. All counts were averaged at each reference point, and all reference points were then averaged to establish an ONL thickness for that particular eye. The counts were performed by three masked observers, and their results were averaged.

### Western blot analysis

Sonicated lysate of mouse retina were collected after centrifugation and further concentrated via lyophilization. Samples consisting of 14 μg protein, as measured by Bradford Protein Assay, were loaded onto a 12% SDS–PAGE gel, and run at 150 V for one hour. The gel was transferred to a PVDF membrane (Immobilon-P, Millipore Corp., Bedford, MA) for 1 h at 200 mA. Membranes were blocked with 1% FBS then incubated with a 1:1000 dilution of anti- glial fibrillary acidic protein (GFAP) IgG1 (Encore Biotech, Inc., Alachua, FL) for 1 h. Membranes were thoroughly washed for subsequent incubation with alkaline phosphatase secondary Ab (Zymed, San Fransisco, CA) for 30 min. Substrate was developed on washed membranes by incubation of 1x BCIP/ NBT dd-H_2_O solution (Zymed). Loading controls were evaluated by stripping membranes at 52 °C in 100 mM 2-mercaptoethanol solution (2% SDS and 63% tris-C, pH 6.7) and washed thoroughly. Blocked membranes were then incubated with 1:1000 diluted α-actin antibody (Sigma, St. Louis, MO), followed by secondary antibody developed in BCIP/NBT solution. Blots were scanned for densitometry analysis using Scion Image software (Scion Corp., Frederick, MD). Each band was assigned a mean density per pixel, which was multiplied by the area of the band selected. This density was then subtracted by the background which was selected within the same lane. The GFAP band (53 kDa) was normalized to the α-actin band (42 kDa) to control for sample loading.

### Histology

Enucleated eyes from freshly euthanized mice (via cervical dislocation) were fixed in 4% paraformaldehyde fixative overnight at 4 °C, followed by a thorough wash in PBS. The samples were submitted (Molecular Pathology Core, University of Florida, Gainesville, FL) for paraffin cross-sectioning at 6 μm and collected every 30^th^ section for staining. These sections were either stained with hematoxylin and eosin, or mounted with Vectashield (Vector Laboratories, Inc., Burlingame, CA) for green fluorescence and DAPI staining visualization via epifluorescence microscopy.

## Results

### Verification of anterior chamber-associated immune induction

In this study, ACAID was induced to BALB/c allosplenocytes via intracameral inoculation on day 0 in C57BL/6 mice. To confirm ACAID induction, hosts received an allomatched subcutaneous immunization on day 7, followed by an allomatched intrapinnal challenge to the ear on day 14. Other mice which received an immunization without prior intracameral inoculation, mounted a strong DTH response, as measured by specific ear swelling post intrapinnal challenge (Figure 1), and thereby indicating induction of systemic immunity. In contrast, the addition of an intracameral inoculation before immunization resulted in the absence of a DTH response, which was similarly observed following intrapinnal challenge of naïve mice, thereby indicating the induction of ACAID.

### Measuring clearance of alloantigen from host anterior chamber following intracameral inoculation

**Figure 3:**
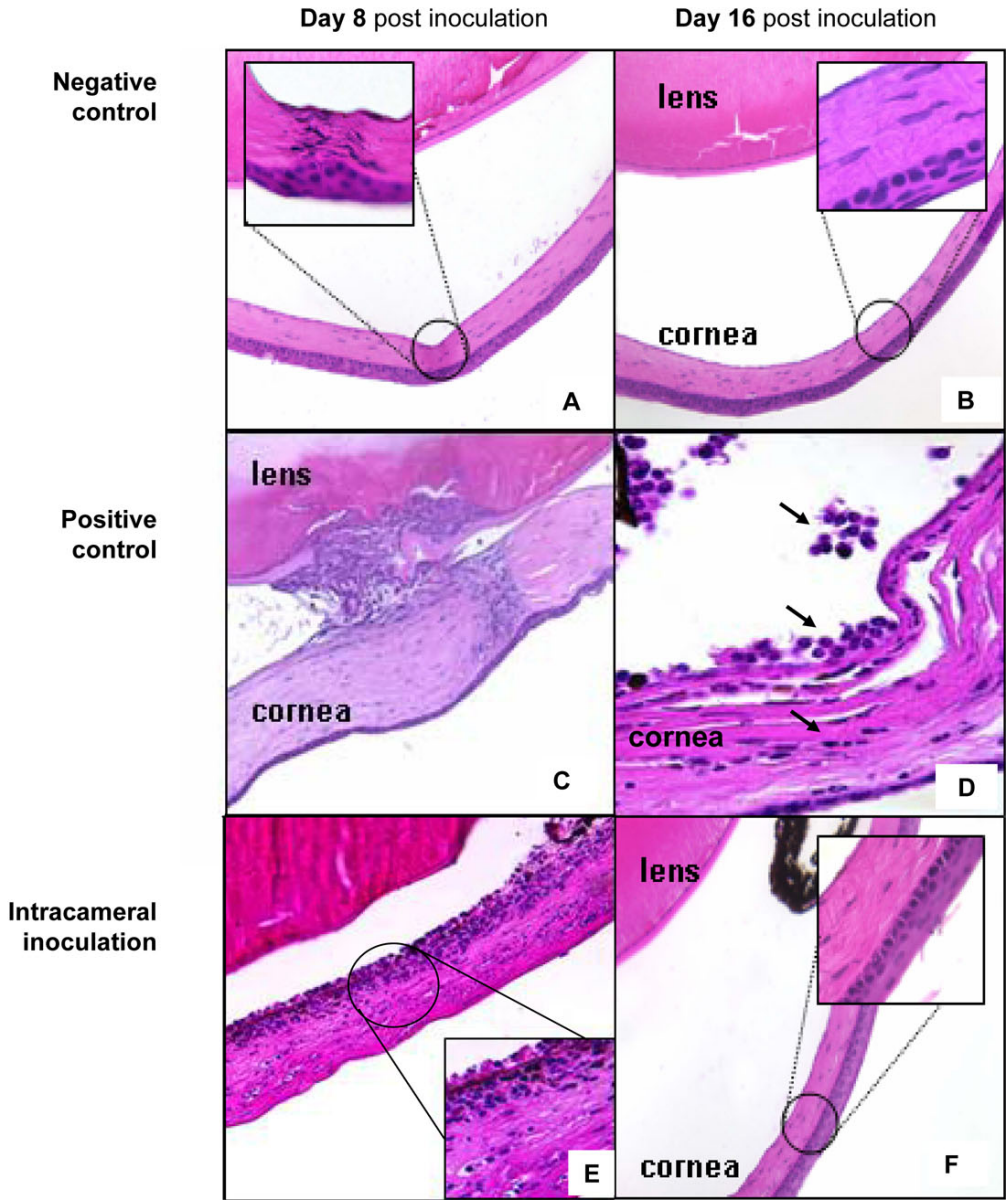
Histopathology of anterior segment tissues following intracameral inoculation. Eyes were histologically examined at various time-points out to 24 days following intracameral inoculation with allosplenocytes (n of 3 per micrograph). Micrographs **(A,B)** represent the histopathology observed in negative control mice eight and 16 days post sham operation. High-magnification image demonstrates marginal scarring observed in these mice **(A**, **inset)**. Micrographs **(C,D)** represent the histopathology observed in positive control mice eight and 16 days post anterior lens puncture. High-magnification image **(D)** demonstrates inflammatory infiltrate in host anterior chamber (a**rrows)**. Micrographs **(E,F)** represent the histopathology observed eight and 16 days post intracameral inoculation with allosplenocytes. High magnification image **(E, inset)** shows inflammatory infiltrate in host posterior cornea, which resolved by day 16 **(F)**.

While clearance of soluble antigens from host anterior chamber (AC) following intracameral inoculation has been well documented, the fate of cellular antigens has received little attention in this regard [36]. We wished to determine the clearance-time of allosplenocytes from the AC and relied on intracameral inoculation and tracking of enhanced green fluorescence protein (eGFP)+ allosplenocytes via epifluorescence microscopy of ocular cross-sections to do so. In performing this experiment we used BALB/c hosts (rather than C57BL/6 hosts used throughout the other parts of this study), and C57BL/6.eGFP(+) mice as donors of the eGFP(+) allosplenocytes. Enucleated eyes were then collected from freshly euthanized mice at 3, 6, 9, 12, and 24 h, as well as 2, 4, and 8 days following intracameral inoculation and evaluated via epifluorescence microscopy. At 3 h post-inoculation, eGFP(+) cells were observed throughout the host AC. By 6 and 9 h, however, eGFP(+) cells were found localized to the iridocorneal angles, presumably exiting the eye via conventional outflow pathways (Figure 2). A small number of eGFP(+) cells were also observed trafficking through the iris and ciliary body as well. Injected cells were mostly absent from host AC by 24 h (Figure 2), as well as the subsequent time points assessed (data not presented).

### Histopathology of anterior segment tissues following intracameral inoculation

Next, we examined anterior segment tissues following intraocular inoculation to identify potential immunopathological changes (such as inflammation and leukocytic infiltration), and the general tempo by which these changes take place. Mice were euthanized at various time points up to 24 days following intracameral inoculation, and freshly enucleated eyes were evaluated histologically. To control for surgical trauma, sham operations were performed by intracameral inoculation with 2 ul sterile HBSS (referred to as the *negative control*). Alternatively, other mice received an anterior-positioned lens puncture (referred to as the *positive control*) as this procedure elicits intracameral inflammation and is thereby an excellent means to positively identify immunopathological changes within the eye [30-33]. Results indicated that negative control mice had little to no pathology, with the exception of marginal scarring of the cornea at the puncture site (Figure 3A,B). In contrast, positive control mice showed significant pathology observed on day 8, which was sustained through day 16 (Figure 3 C,D) and day 24 (day 24 from all groups are not presented). Histological examination revealed anterior synachiae, and leukocytic infiltration of the lens epithelium, anterior chamber, and throughout the cornea. Synachiae was mostly resolved by day 16, while persistence of a substantial infiltrate continued out to at least day 24. Interestingly, intracameral inoculations resulted in anterior segment injuries as well, despite the induction of ACAID confirmed by DTH suppression (Figure 1). Anterior segment injuries were characterized by the histological presence of inflammation and edema of the cornea on day 8 with leukocytic infiltration of the posterior corneal stroma and endothelium (Figure 3E). This pathology was mostly resolved by day 16 (Figure 3F).

### Measuring photoreceptor degeneration following intracameral inoculation

**Figure 4:**
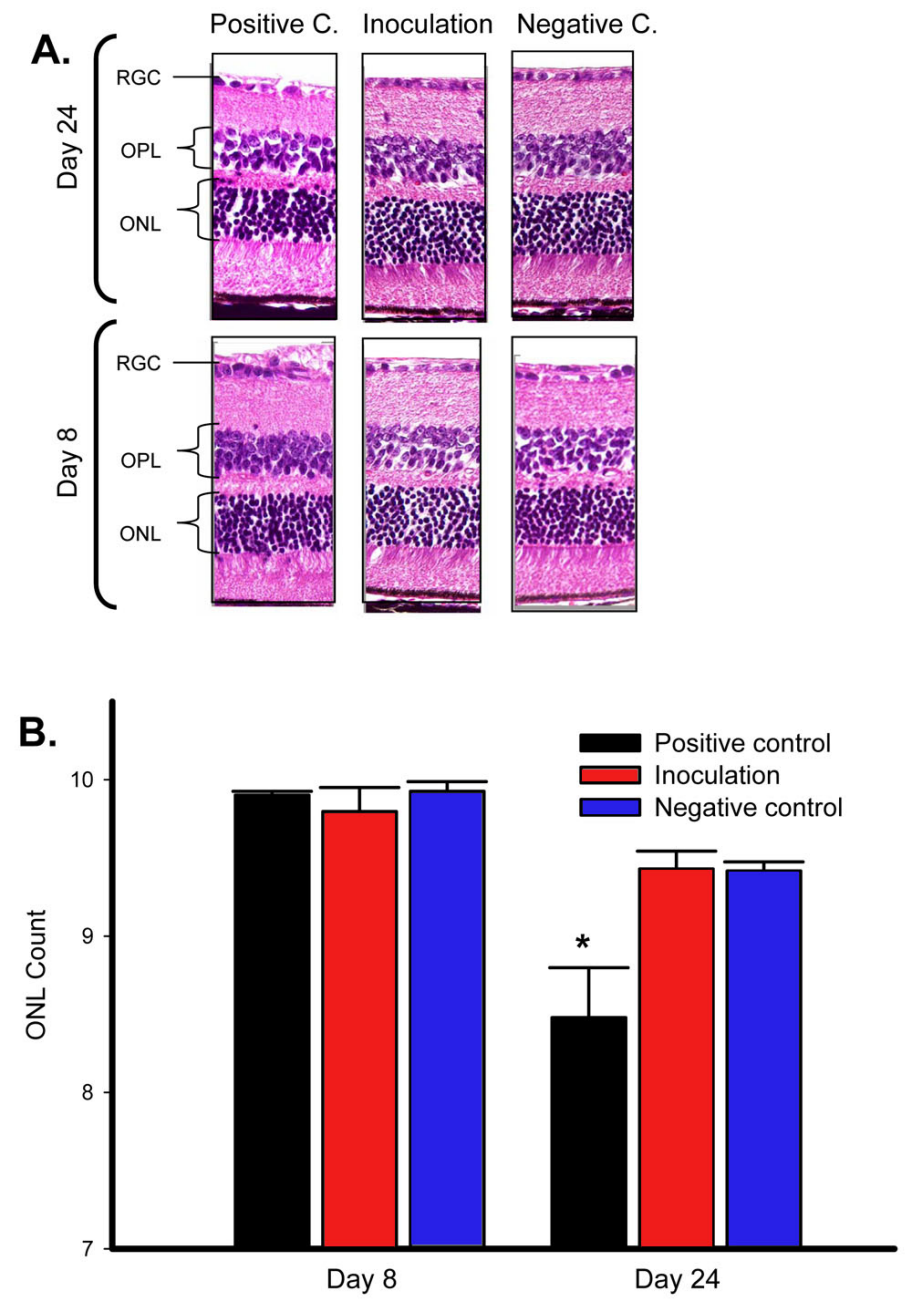
Photoreceptor degeneration is not detected following intracameral inoculation. Inoculation of mice with allosplenocytes intracamerally was compared to positive control (positive c.) and negative control (negative c.) mice. Outer nuclear layer (ONL) thickness was measured histologically over time to identify potential photoreceptor degeneration (n of 7 per micrograph). Representative micrographs of neuroretinal cross-sections include the ganglion cell layer (RGC), outer plexiform layer (OPL), and outer nuclear layer (ONL). Micrographs demonstrate a loss in ONL thickness in positive control mice only. **A:** These data are summarized **(B)** in the adjacent bar graph. Asterisk (*) indicates *p* value ≤ 0.02, as calculated by ANOVA.

As in cystoid macular edema in uveitis, intracameral inflammation can be associated with injury or dysfunction of the neuroretina and can culminate in the degeneration of highly sensitive photoreceptor cells [37]. Because inflammation of anterior segment tissues was confirmed following intracameral inoculation, we wished to further characterize this pathology by determining if photoreceptor cells were degenerating as well. This was assayed by counting the number of adjacent photoreceptor cell nuclei residing in the ONL and measuring the thickness in the ONL over time. As such, positive control mice exhibited a significant 14% loss in ONL thickness relative to the negative control (Figure 4). Interestingly, intracameral inoculations did not result in a significant drop (3%) relative to the negative control mice. Therefore, these data indicate that intracamerally inoculated mice do not undergo photoreceptor degeneration.

### Measuring reactive gliosis following intracameral inoculation

**Figure 5:**
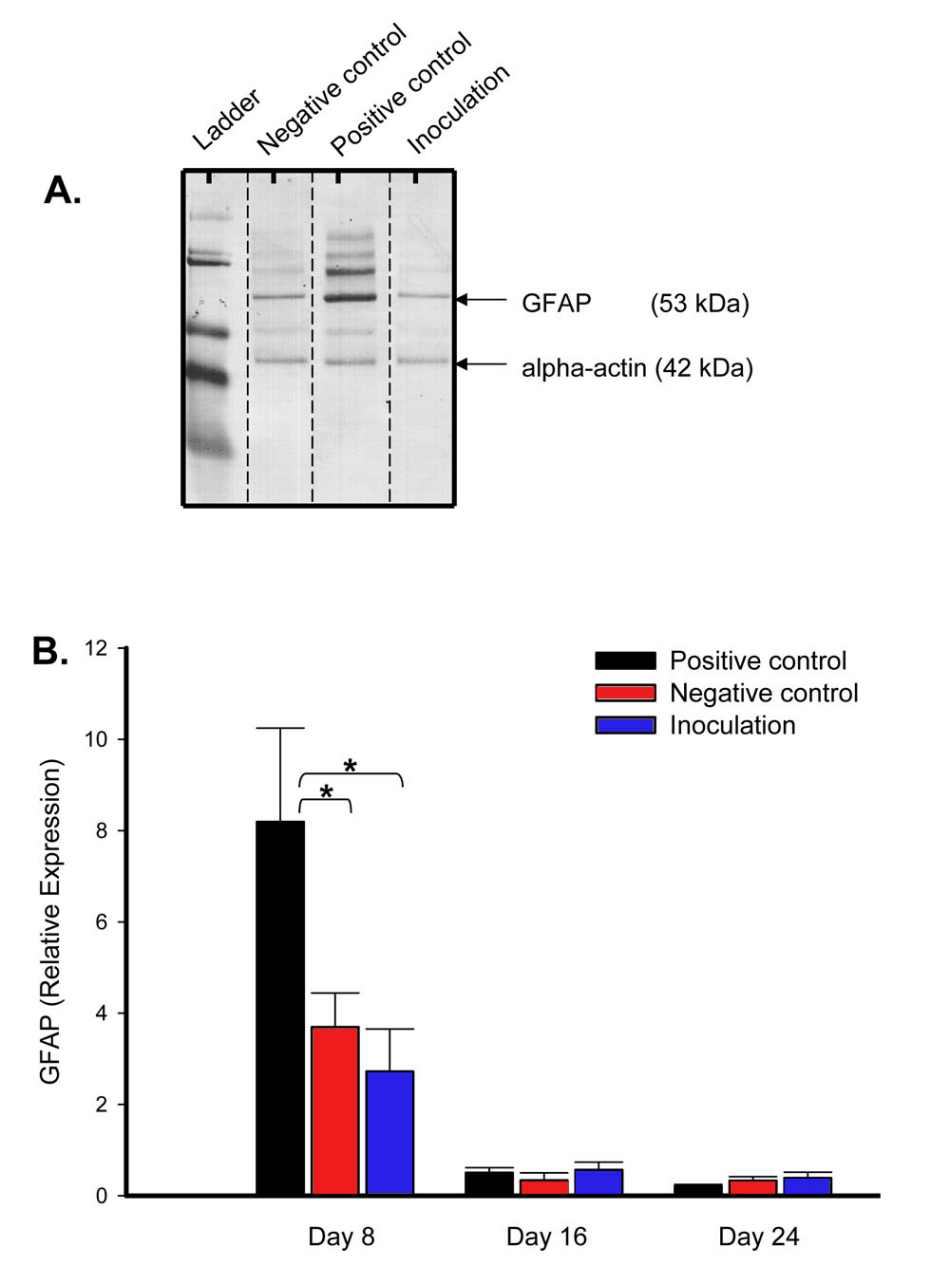
Reactive gliosis is not detected following intracameral inoculation. Neuroretinal sonicate were collected out to day 24 and assessed for glial fibrillary acidic protein (GFAP) expression via western blot analysis (n of 3 neuroretina time-point). A representative western blot from day 8 demonstrates increased GFAP expression observed in positive control mice only.  **A:** Averaged relative densities of respective GFAP bands (53 kDa) normalized to alpha-actin (42 kDa) loading control is summarized in the bar graph. **B:** Levels of GFAP expression in normal eyes are not presented (0.22 – 0.54 relative expression units, n of 4). Asterisk (*) indicates *p* value = 0.01, as calculated by ANOVA.

The absence of photoreceptor degeneration following intracameral inoculation does not eliminate the possibility for presence of other neuroretinal injuries in these mice. Reactive gliosis is another indicator of neuroretinal injury, identified by the upregulation of glial fibrillary acidic protein (GFAP) expression by Muller as well as astrocyte cells. To determine if mice undergo reactive gliosis following intracameral inoculation, we measured retinal levels of GFAP expression by western blot analysis. This was performed with a housekeeping protein α-actin (42 kDa) as a loading control, and densitometry readings of the appropriate bands (53 kDa). Positive control mice resulted in a twofold increase in GFAP expression on day 8 over the negative control (Figure 5). However, intracamerally inoculated hosts did not demonstrate a significant increase of GFAP expression on day 8 over the negative control. For all experimental groups, levels then returned to baseline and were sustained within the range of untreated eyes by day 16 (0.22 to 0.54 relative density units, data not presented).

### Electroretinogram response identifies neuroretinal dysfunction following intracameral inoculation

**Figure 6:**
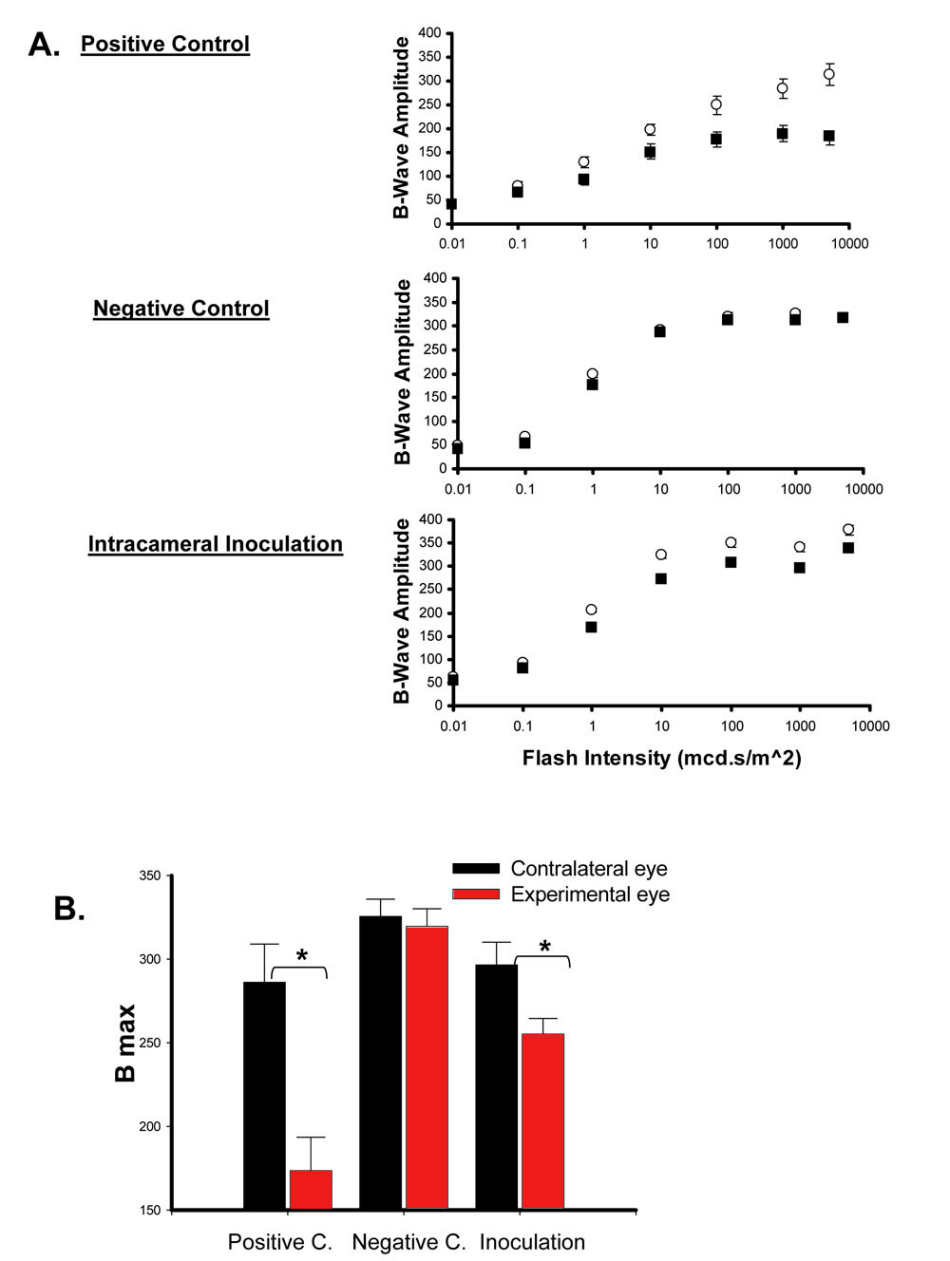
Detectable loss in electroretinogram following intracameral inoculation. Positive control (positive c.), negative control (negative c.), and inoculated mice were compared. Full-field scotopic ERG examinations were performed on treated eyes (■) versus untreated contralateral eyes (◯) on day 8 post inoculation **(A)**. ERG responses were evaluated by mean b-wave amplitudes (uVolts) at each flash intensity (0.01 mcd⋅s/m^2^ to 10000 cd⋅s/m^2^). B max values calculated from nonlinear regressions used for Mann-Whitney U statistical analysis **(B)**. Asterisk (*) indicates a *p* value ≤ 0.05.

Multifocal ERG is an exceptionally sensitive method to identify retinal dysfunction, and is therefore an invaluable clinical tool particularly in subtle and early diagnoses of retinal pathologies. Alternatively, we employed full-field scotopic ERG at increasing flash intensities for examination of b-wave amplitudes in mice out to 24 days post-inoculation. The contralateral untreated eye in each mouse served as an internal control. Results revealed that positive control mice clearly exhibited a sustained loss in ERG responses as compared to their contralateral control (Figure 6A), which was evident on day 8 and sustained through at least day 24 (data not presented). In contrast, negative control mice exhibited no detectable loss in ERG responses as compared to their contralateral untreated eyes. Interestingly, mice receiving intracameral inoculations exhibited a drop in ERG activity on day 8 only, as indicated by a reduction in B-wave amplitudes (Figure 6A). B max values calculated from B-wave amplitudes (Figure 6B) verified the statistical significance of ERG reduction relative to the contralateral eye (p≤0.05). ERG levels in these mice returned to those of the normal eye by days 16 and 24.

## Discussion

With the understanding that ACAID induction is circumvented by select antigens which elicit deleterious local inflammation following intracameral inoculation, we have now shown that antigens (i.e., allosplenocytes) which result in ACAID induction can incur pathological and functional changes as well, even though local inflammation is highly controlled [29]. Our results, therefore, suggest three important points: following intracameral inoculation for ACAID induction (i) the presence/persistence of inflammatory cells is associated with transient, mild retinal dysfunction (as noted in the ERG); (ii) the large majority of antigen is cleared from the AC rather quickly and seems to play a role in controlling intracameral inflammation; and (iii) resulting leukocyte infiltrate can persist in anterior segment tissues well after the bulk of inciting antigen has been cleared, and this delayed presence may play a role in maintaining immune privilege following intracameral inoculation.

In this study, we directly compared intracamerally inoculated mice to lens-punctured mice—an experimental procedure known to elicit robust intracameral inflammation and thereby incorporated here as a means to positively identify immunopathological changes within the eye [30-33]. Sham-operated mice were included as well. Lens puncture releases potent autoantigens such as α-crystallin—a small heat shock protein capable of upregulating nitric oxide, prostaglandins, tumor necrosis factor-α, interleukin (IL)-1, IL-6, and free radicals [30-33]. By administering an AC-positioned puncture, we have shown that significant injury arising in the anterior segment can also result in neuroretinal defects. This was somewhat anticipated, particularly since clinical cases of advanced intracameral inflammation can be associated with concurrent or subsequent injury of the neuroretina (such as cystoid macular edema in uveitis). Our current data support these observations, and this included evidence that anterior lens puncture stimulates inflammatory cell infiltration throughout anterior segment tissues (e.g., lens epithelium, anterior chamber, and cornea) which is sustained to at least day 24. This also correlated with neuroretinal trauma evidenced by a sustained loss in ERG function, increased GFAP levels indicative of active Muller as well as astrocyte cell gliosis, and significant photoreceptor cell degeneration.

Similar to lens-puncture, we show that induction of ACAID following intracameral inoculation of allogeneic splenocytes can also negatively affect the intraocular tissues, albeit less severe and self-limiting in nature. Acute inflammation observed in these mice was characterized by corneal edema and leukocytic infiltration of the posterior corneal stroma and endothelium 8 days post inoculation, as indicated histologically. The apparent focus of the infiltrate observed in the posterior cornea could be anatomically driven, since the AC, which acts as the antigen depot lays directly adjacent to the corneal endothelium. Regarding the neuroretina, intracameral inoculation led to a modest and transient reduction of ERG function signified by a loss in B-wave amplitudes eight days post inoculation—a time point consistent with the identification of intracameral inflammation. However, there was an absence of substantial damage to the neuroretina in these mice, as evidenced by both photoreceptor cell integrity and GFAP levels remaining identical to those of sham-operated control mice. This indicates why ERG reductions as a consequence of intracameral inoculation proved significantly less pronounced than those for mice receiving a lens puncture wound

Several observations made in this study suggest potential mechanisms for controlling the local pathogenesis triggered by allosplenocytes following intracameral inoculation. One mechanism involves the rapid removal of the bulk of antigen from the anterior segment via the iridocorneal angle, signifying the importance of conventional aqueous outflow pathways in controlling intracameral inflammation. A minority of cells was also found exiting through the iris and ciliary body, indicating a role for unconventional outflow pathways as reported by Camelo et al. as well [36]. Even fewer were cells and cell fragments that remained after 24 h. This possibly indicates antigen processing by resident myeloid cells such as F4/80+ antigen presenting cell (APC), or apoptosed cells via Fas-FasL, both of which are important intracameral activities in the induction of ACAID [6,12,38]. Be that as it may, we also observed the presence of morphologically intact injected cells (eGFP+) exiting from the AC, and nonetheless verified that ACAID is induced following intracameral inoculation with allosplenocytes. This possibly highlights the exceptional capacity of immune privileged sites to maintain low affinity environments for allostimulation due, in part, to the presence of immature APC largely devoid of MHC and costimulatory molecule expressions within the cornea.

Another important observation made here which may also play a role in controlling the immunopathology following intracameral inoculation involves the presence of inflammatory cells in anterior segment tissue, even after inciting antigen has been cleared. While seemingly counterintuitive, the local recruitment as well as the persistence of inflammatory cells may actively be involved in the maintenance of immune privilege following intracameral inoculation. Sohn et al. showed that the ligation of a C3 complement activation factor (iC3b), which is constitutively present in aqueous humor, stimulates APC upregulation of IL-10 and TGF-β —two immunosuppressive cytokines critical for ACAID induction [39]. Furthermore, APC are also capable of activating latent TGF-β, which is also constitutively present in aqueous humor. Thus, by increasing the concentrations of IL-10 and TGF-β, the recruitment of APC to the anterior segment can support the maintenance of an immunosuppressive microenvironment. This point favors the notion that intracameral inflammation is actively involved in ACAID induction following intracameral inoculation; this potentially novel mechanism requires further investigation. Nevertheless, a lack of infiltration within the anterior segment of sham-operated controls suggests that inflammation in the eyes of experimental mice is dependent on the presence of allogeneic cells and is not a consequence of the injection per se.

In summary, we show that ACAID induction following intracameral inoculation can result in intraocular injury in the form of anterior segment inflammation and ERG dysfunction—an immunopathology that is markedly reduced and transient when compared to a more serious lens-puncture wound. The importance of these findings is relevant to our fundamental understanding of in vivo generation in ACAID induction. Our findings are also relevant in studies that utilize this form of ACAID induction as a readout to assess parameters of immune privilege in experimental models such as corneal transplantation and neovascularization, uveitis, and glaucoma [24-26]. In addition, because tolerance induction via intracameral inoculation can effectively ameliorate immune-mediated pathologies in several rodent models (i.e., allograft rejection, autoimmune uveoretinitis/encephalomyelitis, and lung hyperreactivity), it is therefore considered to be an attractive strategy for clinical use [2,3,21-23]. In this regard, our data indicate that careful consideration should be given when selecting potential candidates for intracameral inoculation, particularly patients with active or a previous history of corneal as well as retinal pathologies.
